# White matter brain age as a biomarker of cerebrovascular burden in the ageing brain

**DOI:** 10.1007/s00406-024-01758-3

**Published:** 2024-02-29

**Authors:** Jing Du, Yuangang Pan, Jiyang Jiang, Ben C. P. Lam, Anbupalam Thalamuthu, Rory Chen, Ivor W. Tsang, Perminder S. Sachdev, Wei Wen

**Affiliations:** 1 Centre for Healthy Brain Aging (CHeBA), School of Psychiatry, UNSW Sydney, Kensington, New South Wales 2052 Australia; 2 Neuropsychiatric Institute (NPI), Euroa Centre, Prince of Wales Hospital, Randwick, NSW 2031 Australia; 3 Centre for Frontier AI Research (CFAR), A*STAR, Singapore, 138623 Singapore; 4 Australian Artificial Intelligence Institute (AAII), UTS, Sydney, NSW 2007 Australia

**Keywords:** Vascular risk factors, White matter brain age, Diffusion weighted imaging, Deep learning networks

## Abstract

As the brain ages, it almost invariably accumulates vascular pathology, which differentially affects the cerebral white matter. A rich body of research has investigated the link between vascular risk factors and the brain. One of the less studied questions is that among various modifiable vascular risk factors, which is the most debilitating one for white matter health? A white matter specific brain age was developed to evaluate the overall white matter health from diffusion weighted imaging, using a three-dimensional convolutional neural network deep learning model in both cross-sectional UK biobank participants (*n* = 37,327) and a longitudinal subset (*n* = 1409). White matter brain age gap (WMBAG) was the difference between the white matter age and the chronological age. Participants with one, two, and three or more vascular risk factors, compared to those without any, showed an elevated WMBAG of 0.54, 1.23, and 1.94 years, respectively. Diabetes was most strongly associated with an increased WMBAG (1.39 years, *p* < 0.001) among all risk factors followed by hypertension (0.87 years, *p* < 0.001) and smoking (0.69 years, *p* < 0.001). Baseline WMBAG was associated significantly with processing speed, executive and global cognition. Significant associations of diabetes and hypertension with poor processing speed and executive function were found to be mediated through the WMBAG. White matter specific brain age can be successfully targeted for the examination of the most relevant risk factors and cognition, and for tracking an individual’s cerebrovascular ageing process. It also provides clinical basis for the better management of specific risk factors.

**Supplementary Information:**

The online version contains supplementary material available at 10.1007/s00406-024-01758-3.

## Introduction

Vascular dementia accounts for at least 20% cases of dementia and is the second leading cause of the cognitive decline following Alzheimer’s dementia [1]. Exposure to different vascular risk factors such as hypertension, diabetes, and hypercholesterolemia aggravate the vascular burden and accelerate the progression to cognitive decline [2]. Some surrogate white matter (WM) lesions are widely used for evaluating the neurovascular health, such as the white matter hyperintensity (WMH), microbleeds, enlarged perivascular spaces [3]. However, some subtle damage of the WM has occurred several decades before these lesions can be observed via magnetic resonance imaging (MRI). While some diffusion weighted imaging (DWI) measures such as fractional anisotropy (FA) and mean diffusivity (MD) can be used to examine the WM microstructural integrity, they typically capture distinct physiological properties [4]. A composite index for evaluating the overall WM health would be necessary to investigate the associations between the different risk factors and the cerebrovascular burden.

Given that different organs or systems usually exhibit heterogeneous ageing rates, an individual might have multiple underlying bodily ages [5], such as bone age, renal age, in addition to their chronological age. Brain age is a special case in this context, and it arguably reflects the brain health. Brain age gap (BAG) is the difference calculated by subtracting the chronological age from predicted brain age. Previous studies have investigated brain age using high-dimensional neuroimaging data for healthy populations [6] or people with specific brain diseases [7, 8]. The three-dimensional convolutional neural network (3D-CNN) deep learning model has been widely applied for brain age prediction due to its high performance and reliability in feature extraction [9, 10], capturing the non-linear and intricate features from the raw imaging hierarchically. To our knowledge, a large body of studies thus far have generated a single brain age using T1-weighted imaging scans. However, associations of vascular risk factors with tissue-specific brain age, especially white matter brain age derived from DWI scans, have not been fully investigated.

The primary objective of this study was to examine the extent of the collective or individual associations between vascular risks and the acceleration of cerebrovascular aging, as quantified by white matter brain age. Using deep learning techniques, we computed a white matter brain age from five DWI-derived maps. The individual and accumulative effects of vascular risk factors and their sex stratifications on the white matter brain age gap (WMBAG) and cognition were examined. We hypothesised that the DWI-derived white matter brain age would reflect the cumulative cerebrovascular burden and the rate of cerebrovascular ageing acceleration was dependent on the specific risk factors.

## Methods

### Participants

**Fig. 1:**
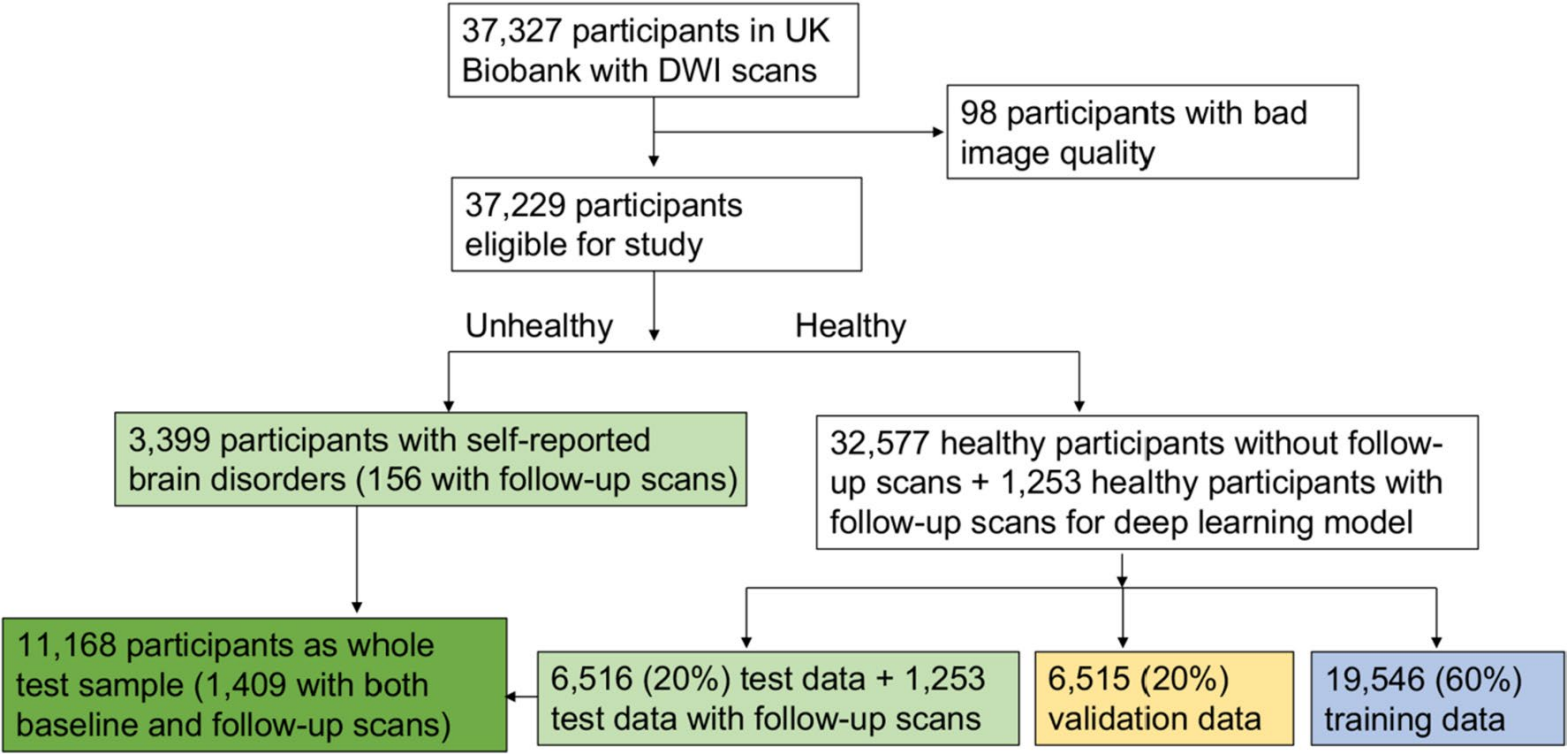
Flowchart of participant selection

Data for this study were drawn from UK Biobank, a large-scale ongoing prospective population-based cohort study [11]. A flowchart of the selection of participants can be found in Fig. 1. Briefly, after visual inspection of 37,327 eligible DWI scans, 98 participants with poor image quality were removed, leaving 37,229 at baseline to be included in this study. The exclusion criteria were: (1) scans with incomplete brain; (2) the presence of severe brain lesions such as the tumours; and (3) distorted scans and/or scans with poor quality due to factors such as the severe head motion, magnetic field inhomogeneity or metallic objects. After excluding 3399 participants with severe self-reported brain related disorders (Field ID 20002, Supplementary Table e-1) to ensure a relatively healthy sample for deep learning training, 60% (*n* = 19,546) of the remaining participants were randomly selected to the training set. Twenty percent (*n* = 6515) were used as the validation set, which provided an unbiased evaluation of a model fit on the training dataset while selecting the model's structures (e.g., the type of loss function). The remaining 20% were combined with the unhealthy participants as identified above (*n* = 11,168) for use in the test set. In this test sample, 1409 participants had both baseline and follow-up scans that were used for longitudinal analysis.

The ethics of this study has been approved by the North West Multi-centre Research Ethics Committee (MREC) and written informed consent was obtained from all participants.

### MRI acquisition and imaging processing

Details of DWI acquisition protocols can be found in the online UK Biobank brain imaging documentation (https://biobank.ctsu.ox.ac.uk/crystal/crystal/docs/brain_mri.pdf). DWI scans were acquired from three imaging centres (Cheadle Greater Manchester, Newcastle and Reading, UK), and each centre used a 3T Siemens Skyra scanner with a standard Siemens 32-channel head coil and same parameters. The original DWI data had been pre-processed with eddy currents, head motion correction and distortion correction by UK Biobank using the FMRIB Software Library (FSL) toolkit [12]. The diffusion-tensor-imaging fitting tool (DTIFIT) was used to generate the following DWI-derived maps in native space: FA (fractional anisotropy), MD (mean diffusivity), AxD (axial diffusivity), RD (radial diffusivity) and MO (tensor mode). All individual maps were nonlinearly warped to a 2 × 2 × 2 mm^3^ MNI-152 standard space using FNIRT (FMRIB’s Nonlinear Image Registration Tool) [13], and were visually inspected and finally used as the input for the deep learning model.

### White matter brain age computation

**Fig. 2:**
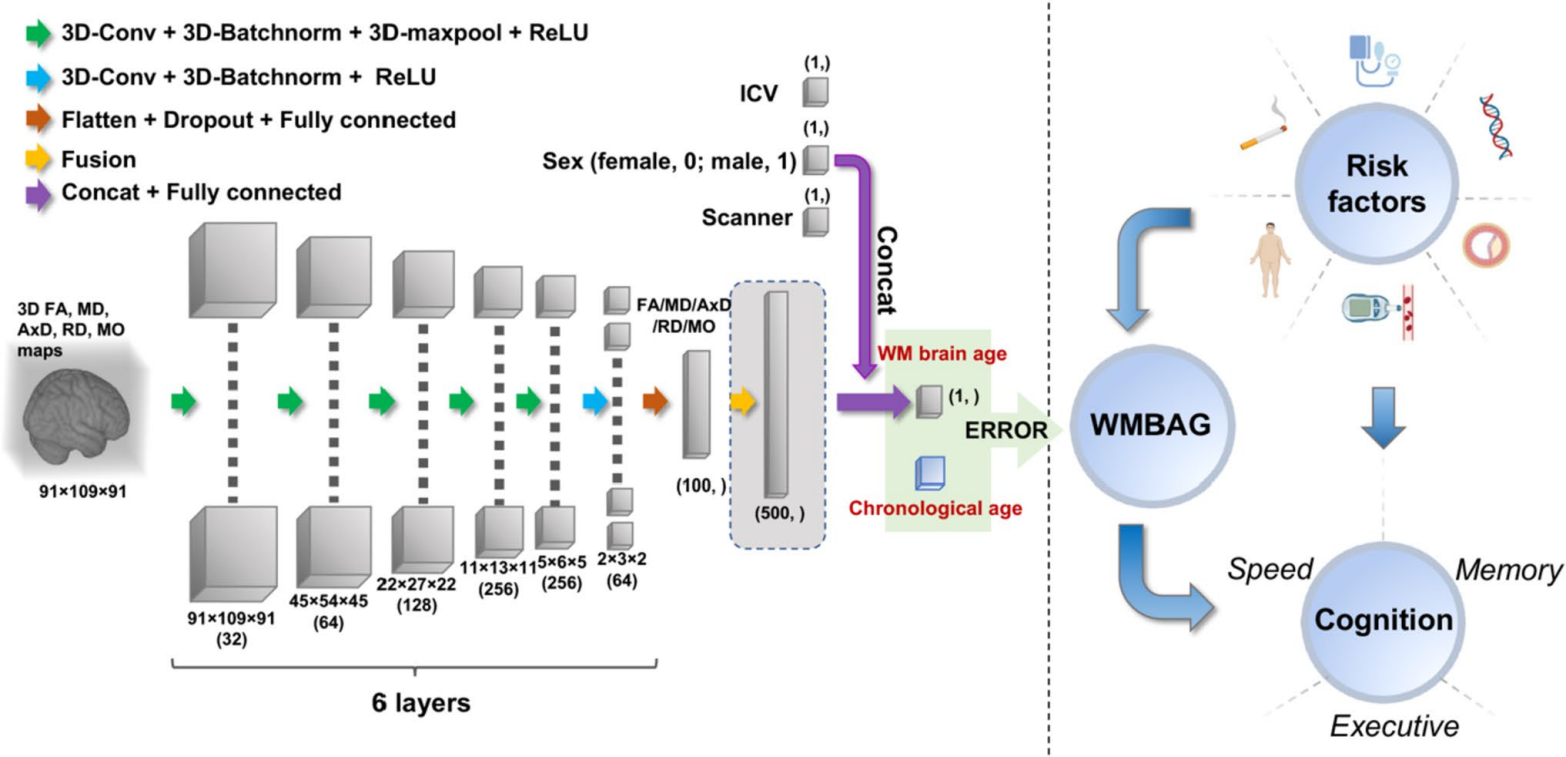
Overview of study design. The left panel shows the 3D convolution neural network architecture; the right panel shows the clinical analyses between risk factors and WMBAG and cognition. Inputs of the model are pre-processed 3D DWI maps, WMBAG = White matter brain age gap. Abbreviations: 3D = three-dimensional; Conv = convolution; Batchnorm = batch normalization; ReLU = rectified linear unit; WM = white matter; WMBAG = white matter brain age gap; FA = fractional anisotropy; MD = mean diffusivity; AxD = axial diffusivity; RD = radial diffusivity; MO = anisotropy mode

A three-dimensional convolutional neural network (3D-CNN) deep learning model was used to establish white matter brain age, the structure of which is illustrated in Fig. 2. The architecture follows the Simple Fully Convolutional Network (SFCN) proposed by Peng Han et al. [14], which is based on VGGNet [15] with fully convolutional structures.

Briefly, the network received a 91 × 109 × 91 3D image and the corresponding sex and scanner of a participant as input, and the output was the predicted age at the last layer. The network consisted of eight blocks, as shown in Fig. 2, and each of the first five blocks contained a 3D convolutional layer with kernel size 3 × 3 × 3, a 3D batch normalisation layer, a 3D max-pooling layer, and a ReLU [16] activation layer. The sixth block had a 1 × 1 × 1 3D convolutional layer, a batch normalisation layer, and a ReLU activation layer. The seventh block contained a dropout layer (activated only during the training process by randomly dropping 50% of the elements) and a fully connected layer. The spatial dimension was reduced to 2 × 3 × 2 after the sixth block. The flatten operator was applied to resize the tensor to a vector before applying the seventh block. Instead of going through the 3D-CNN network as images, the extra information such as sex and scanner was incorporated into the feature map by concatenation before the eighth block. Finally, linear regression was employed in the eighth block for fusing the image features and the information of sex and scanner, with the output being a scalar for the predicted white matter brain age. The channel numbers used in the first six 3D convolution layers were [32, 64, 128, 256, 256, 64].

The internal process of the model can be summarised into three stages: (1) *Nonlinear feature extraction*: The first six blocks extracted feature maps from each input image; (2) *Tensor to vector*: The seventh block smoothly transformed the 3D tensor to a vector for downstream age prediction; and (3) *Linear regression*: The eighth block incorporated the extra sex and scanner information, and the output was the predicted age.

#### Network architecture for fusing all five diffusion maps

To increase the white matter brain age prediction accuracy, the five resultant DTI maps (FA, MD, AxD, RD and MO) were incorporated to generate a composite metric. Five 3D CNN networks with the same structure as discussed above were applied, with each network modelling each feature map separately. In terms of feature fusion, we adopted a simple concatenation to fuse the five feature maps after the seventh block, as well as the covariates (i.e., sex, scanner and ICV). The three covariates were applied to all five feature maps. The resultant feature map was a vector with (100 * 5 + 3) entries, namely 100 entries for each feature map and 3 entries for covariates. Similarly, linear regression was employed in the eighth block for mapping the fused features into the final predicted age.

To reduce the computational cost, instead of retraining five 3D CNN networks simultaneously from scratch, we reused the intermediate feature maps learned during the analysis of each of the five DWI-derived maps and only trained the eighth block accordingly.

#### Bias correction

The predicted ages normally suffer from the issue of underfitting due to regression dilution and non-Gaussian age distribution, which means older participants will be estimated with a younger brain age while younger participants will be estimated with an older brain age. As reported by Smith et al. [17], bias correction is an essential postprocessing technique in most brain-age prediction studies. $y$ and $\hat{y}$ denote the chronological age and predicted age, respectively. We can fit a linear regression $\hat{y} = \alpha y + \beta$ on the left-out validation set with known chronological age. Applying the learned coefficients $\left( {\alpha ,\beta } \right)$, the corrected predicted age $\hat{y}_{co}$ for test set can be estimated by

$$\hat{y}_{co} = \frac{{\hat{y} - \beta }}{\alpha }$$

where we assume the coefficients $\left( {\alpha ,\beta } \right)$ can be generalised to the test set.

#### Model performance

Model performance was evaluated by two predominant measures in this study. Mean absolute error (MAE) was defined as $MAE = \frac{1}{n}\sum_{i = 1}^n {\left| {predicted\_age_i - chronological\_age_i } \right|}$, Pearson’s correlatin coefficient (Pearson’s r) was applied to characterise the correlation between chronological age and predicted age.

### Vascular risk score (VRS) and apolipoprotein E (APOE) ε4 carrier status

We incorporated five essential vascular risk factors, i.e.: (1) hypertension; (2) diabetes; (3) hypercholesterolemia; (4) obesity; and (5) smoking. Each vascular risk factor was binarised with 1 indicating presence of that factor and 0 otherwise. A composite vascular risk factor score (VRS) was generated to evaluate the overall cerebrovascular burden by calculating the total numbers of vascular risk factors using a method applied similarly in other studies [18]. Given that there were a very small number of subjects who scored 4 (*n* = 408, 3.7%) or 5 (*n* = 82, 0.7%), the VRS was categorised into 0, 1, 2 and ≥ 3. APOE ε4 carrier status was classified into three categories based on the number of ε4 alleles, i.e., non-carriers; carriers with one or two ε4 allele(s). Details of evaluation of vascular risk factors were summarised in Supplementary Methods.

### Cognitive tests

Seven neuropsychological tests were included: Reaction Time, Trail Making Test A and Symbol Digit Substitution for assessing *processing speed*; Numeric Memory and Pairs Matching for assessing *memory*; Trail Making Test B and Fluid Intelligence for assessing *executive function*. Further details of the standardisation procedure can be found in our previous work [19] or Supplementary Methods.

### Statistical analysis

Statistical analyses were conducted using SPSS version 26.0 and R version 3.6.1. Two-tailed *p* < 0.05 was considered statistically significant. Continuous variables were described as mean ± SD (standard deviation); categorical or binary variables were described as number and percentage. The difference of WMBAG between healthy and unhealthy participants in the baseline test set was compared using Analysis of Covariance (ANCOVA) adjusting for chronological age, sex, scanner and APOE status.

Multiple linear regression models were conducted to investigate the associations between vascular risk factors and WMBAG at baseline. VRS was dummy coded with the 0 category as reference in all analyses. Eight regression models were used in this analysis for analysing the vascular risk factors—see their mathematical expressions in Supplementary methods. For model 1a, we investigated the main effect of VRS on the white matter brain age gap. For model 1b, interaction terms (sex times different VRS levels) were added to the model to examine if the effects of VRS differ by sex. In model 2a, to determine the specific contribution of each risk factor, the VRS was replaced with all five vascular risk factors. In model 2b–f, we investigated the moderation effect of sex on the relationship between each vascular risk factor and WMBAG. Chronological age, sex, scanner and APOE status were controlled for all models.

The association between WMBAG and cognition at baseline was first examined. Then mediation analysis with WMBAG as a mediator and cognition as outcome, was carried out among baseline participants with VRS and individual risk factors as predictors. Baseline chronological age, sex, scanner, APOE and education were controlled. Bonferroni correction was applied for these analyses with four cognitive outcomes (corrected alpha level = 0.05/4 = 0.0125). Mediation analysis was performed using the ‘mediation’ package [20] in R. Direct and indirect effects were estimated via bootstrapping with 5000 samples.

For longitudinal analysis, a dependent t-test was conducted to examine change in WMBAG between baseline and follow-up. To explore the prospective effects in the longitudinal subset, we first conducted multiple linear regression to examine the relationships between baseline vascular risk factors and change in WMBAG (calculated as the difference between follow-up and baseline scores), and that between WMBAG change and cognition change. Mediation analysis was then conducted to examine the direct and indirect effects of vascular risk factors on change in cognition, through change in WMBAG.

### Data and code availability statement

The UK Biobank data can be accessed by online application (https://www.ukbiobank.ac.uk/). Codes for the 3D-CNN deep learning model in this study can be shared from the authors upon request.

## Results

### Sample characteristics

Sample characteristics including demographics and vascular risk factors of test data are shown in Table 1. Cross-sectional test data included 7769 healthy participants and 3399 unhealthy participants. Among them, 1409 participants with both the baseline and follow-up scans were used for longitudinal analysis.

**Table 1:** Characteristics of test samples

	Cross-sectional test sample (instance 2)			Longitudinal test sample (baseline instance 2, follow-up instance 3)	Follow-up (*n* = 1409)
	All test data (*n* = 11,168)	Healthy test data (*n* = 7769)	Unhealthy test data (*n* = 3399)	Baseline (*n* = 1409)	
*Demographics*					
Male, number (%)	5111 (45.8)	3721 (47.9)	1390 (40.9)	685 (48.6)	–
Education, college number (%)	5434 (49.1)	3779 (49.1)	1655 (49.1)	684 (48.9)	–
Chronological age, years, mean ± SD range (min, max)	63.94 ± 7.52 (45.49, 82.32)	64.21 ± 7.45 (45.49, 82.32)	63.30 ± 7.62 (45.93, 80.97)	63.05 ± 7.17 (47.01, 80.33)	65.30 ± 7.17 (49.36, 82.61)
*Risk factors*					
Hypertension, number (%)	5618 (50.4)	3858 (49.8)	1760 (51.8)	703 (49.9)	–
Diabetes, number (%)	610 (5.5)	390 (5.0)	220 (6.5)	66 (4.7)	–
Hypercholesterolemia, number (%)	2713 (24.5)	1706 (22.2)	1007 (30.0)	299 (21.4)	–
Obesity, number (%)	2070 (19.0)	1265 (16.8)	805 (23.9)	232 (16.6)	–
Smoking, number (%)	4222 (38.1)	2863 (37.2)	1359 (40.3)	459 (32.8)	–
*VRS, number (%)*					
Score = 0	2659 (24.7)	1935 (26.1)	724 (21.8)	400 (28.9)	–
Score = 1	3718 (34.6)	2654 (35.7)	1064 (32.0)	496 (35.8)	–
Score = 2	2600 (24.2)	1756 (23.6)	844 (25.4)	290 (20.9)	–
Score ≥ 3	1772 (16.5)	1082 (14.6)	690 (20.8)	200 (14.4)	–
*APOE ε4 carrier status, number (%)*					
Non-carrier	6739 (72.2)	4728 (72.2)	2011 (72.2)	839 (70.7)	–
Carrier with one ε4 allele	2379 (25.5)	1666 (25.5)	713 (25.6)	321 (27.1)	–
Carrier with two ε4 alleles	210 (2.3)	150 (2.3)	60 (2.2)	26 (2.2)	–

Instance 2/3 means the first or second imaging assessment. Due to some missing values of the risk factors, the valid percentage of the risk factors were calculated for the remaining participants. The numbers for cross-sectional analysis are: college, *n* = 11,071; hypertension, *n* = 11,151; diabetes, *n* = 11,103; hypercholesterolemia, *n* = 11,060; obesity, *n* = 10,880; smoking, *n* = 11,073; VRS, *n* = 10,749; APOE status, *n* = 9328

*SD* standard deviation; *VRS* vascular risk score; *APOE* Apolipoprotein E

### White matter brain age prediction

**Fig. 3:**
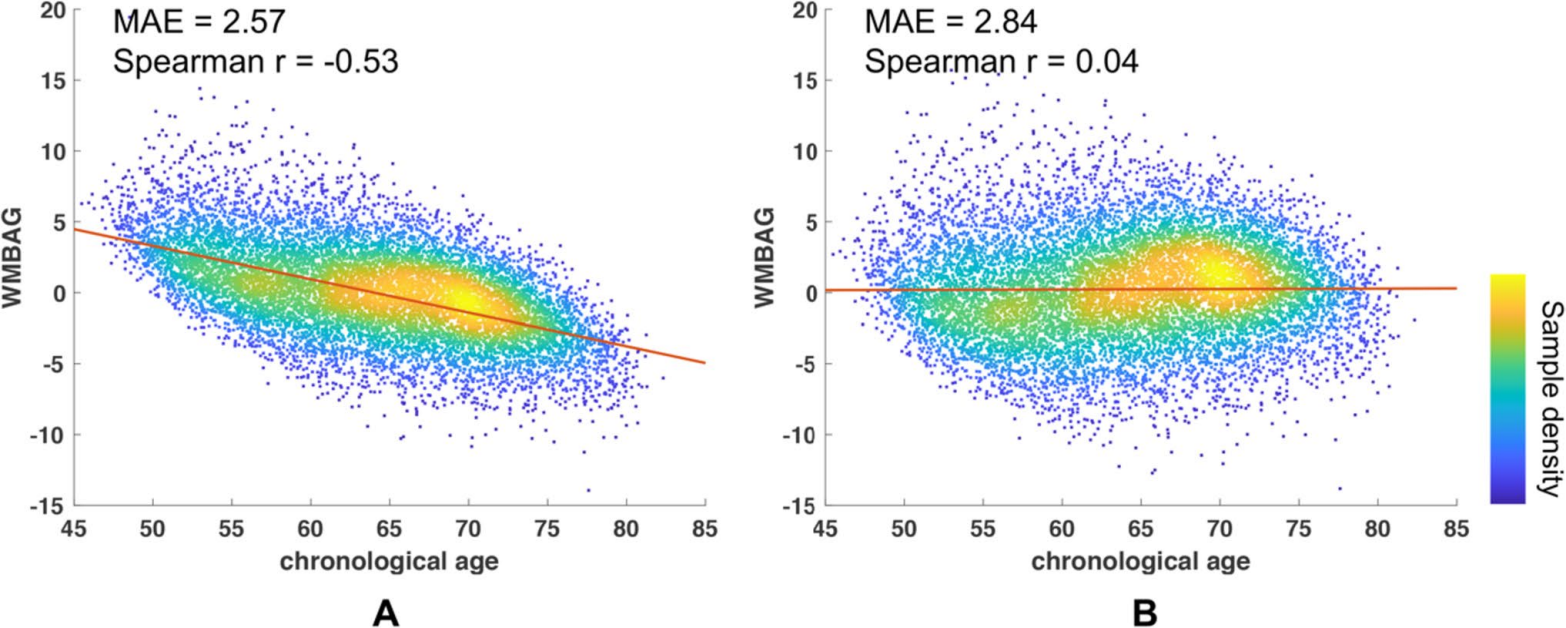
Bias correction. Association between chronological age and uncorrected WMBAG (**A**); Association between chronological age and bias-corrected WMBAG (**B**). All cross-sectional test subjects were included in this bias correction analysis (*n* = 11,168). MAE and the correlation coefficient (r) are listed in the upper left corner of each sub-plot. Colour bar indicates the sample density. WMBAG = White matter brain age gap. Abbreviations: WMBAG = white matter brain age gap; Spearman r = coefficient for Spearman correlation; MAE = mean absolute error; WM = white matter

The white matter brain age predictions before and after bias correction for the whole test set are shown in Fig. 3A and B. Spearman correlation coefficient between WMBAG and chronological age for the whole cross-sectional test participants was reduced from − 0.54 before bias correction to 0.04 after bias correction, with a slight increase of MAE from 2.57 to 2.84. Pearson’s r between white matter brain age and chronological age is 0.902.

Cross-sectional white matter brain age which was computed using our 3D-CNN model and WMBAG are summarised in Table 2. Interestingly, participants with none of the vascular risk factors had a negative mean WMBAG of 0.56, which suggested that they had a brain 0.56 years younger on average than their chronological age. The MAE for the fused white matter brain age was smaller than that of any other single DWI derived map (Supplementary Table e-2). MAE measured on the healthy test data was 2.75 years (Supplementary Table e-2 and Figure e-1A) with Pearson’s r between chronological and predicted brain age of 0.908 (*p* < 0.001). For unhealthy test data (Supplementary Table e-2 and Figure e-1B), the MAE was 3.03 with Pearson’s r = 0.892 (*p* < 0.001). Due to the best performance of the fusion of all five DWI maps, we conducted the subsequent clinical analysis using the fused predicted age. The mean WMBAG for unhealthy test participants was 0.51 ± 0.08 years older than healthy test participants (*p* < 0.001, 95% CI = 0.348–0.668).

**Table 2:** White matter brain age and WMBAG

	White matter brain age, years, mean ± SD range (min, max)	WMBAG, years, mean ± SD range (min, max)
*Cross-sectional test sample (instance 2)*		
All (*n* = 11,168)	64.17 ± 8.35 (45.13, 84.06)	0.23 ± 3.60 (− 13.82, 20.91)
Healthy test data (*n* = 7769)	64.30 ± 8.31 (45.13, 83.60)	0.09 ± 3.49 (− 12.50, 13.94)
Unhealthy test data (*n* = 3399)	63.86 ± 8.45 (45.73, 84.06)	0.56 ± 3.82 (− 13.82, 20.91)
*Longitudinal test sample (baseline instance 2, follow-up instance 3)*		
Baseline (*n* = 1409)	62.94 ± 8.02 (45.71, 81.77)	− 0.11 ± 3.45 (− 12.25, 13.59)
Follow-up (*n* = 1409)	65.28 ± 8.05 (46.02, 82.98)	− 0.02 ± 3.45 (− 12.46, 12.90)
*VRS levels for all test data*		
Score = 0 (*n* = 2659)	60.57 ± 7.91 (45.13, 82.23)	− 0.56 ± 3.53 (− 12.06, 20.91)
Score = 1 (*n* = 3718)	63.30 ± 8.15 (45.73, 83.22)	− 0.02 ± 3.55 (− 12.72, 15.70)
Score = 2 (*n* = 2600)	66.16 ± 7.88 (46.26, 84.06)	0.64 ± 3.54 (− 13.82, 14.68)
Score ≥ 3 (*n* = 1772)	68.19 ± 7.41 (47.01, 83.60)	1.29 ± 3.58 (− 10.94, 14.79)

Instance 2/3 means the first or second imaging assessment

*WMBAG* white matter brain age gap; *SD* standard deviation; *VRS* vascular risk score

### Cross-sectional analysis at baseline

#### Associations between risk factors and WMBAG

**Fig. 4:**
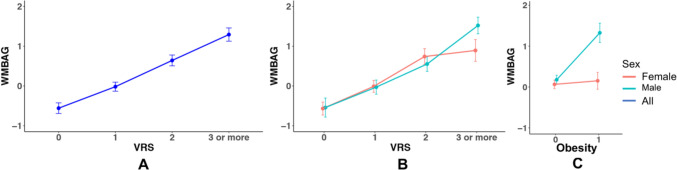
WMBAG across different VRS groups (**A** for all participants; **B** for males and females separately) and WMBAG for different obesity status by sex (**C**). Each dot indicates the mean value for the WMBAG, error bar indicated the 95% CI. Abbreviations: WMBAG = white matter brain age gap; VRS = vascular risk score; WM = white matter. CI = confidence interval

In model 1a, after controlling for chronological age, sex, scanner and APOE status, participants with one, two, and three or more vascular risk factors had an increased WMBAG of 0.54, 1.23, and 1.94 years older, respectively, than those without vascular risk factors (Table 3; also see Fig. 4A). In model 1b, significant interaction between sex and VRS on its association with WMBAG was found (*p* = 0.015; Table 3). Among participants with three or more vascular risk factors, the WMBAG of males was significantly larger than that of females (mean difference = 0.617 years, *p* = 0.001). No significant difference of WMBAG between males and females was found for those with 0, 1 or 2 risk factors (Fig. 4B).

**Table 3:** Association between VRS and WMBAG

	Unstandardised beta	95% CI		*p*-value
		Lower bound	Upper bound	
*Main effect (model 1a)*				
Chronological age	− 0.026	− 0.036	− 0.016	< 0.001
Sex	0.087	− 0.063	0.237	0.258
Scanner	0.231	0.145	0.317	< 0.001
APOE status	0.047	− 0.098	0.192	0.528
VRS_1	0.538	0.345	0.730	< 0.001
VRS_2	1.229	1.014	1.444	< 0.001
VRS_3	1.936	1.692	2.181	< 0.001
*Interactions (model 1b)*				
Chronological age	− 0.026	− 0.036	− 0.016	< 0.001
Sex	0.01	− 0.299	0.318	0.951
Scanner	0.232	0.146	0.318	< 0.001
APOE status	0.049	− 0.096	0.194	0.507
VRS_1	0.543	0.301	0.785	< 0.001
VRS_2	1.282	1.000	1.564	< 0.001
VRS_3	1.572	1.217	1.928	< 0.001
VRS_1 * sex	0.002	− 0.395	0.400	0.991
VRS_2 * sex	− 0.076	− 0.505	0.354	0.730
VRS_3 * sex	0.607	0.119	1.095	0.015

Main effect of VRS on WMBAG was analysed by recoding VRS into dummy variables as independent variables. Interaction effects were analysed by adding their corresponding interaction terms to the model

*WMBAG* white matter brain age gap; *VRS* vascular risk score; *APOE* Apolipoprotein E; *CI* confidence interval, *VRS_1* is the dummy variable indicating participants with only 1 vascular risk factor; *VRS_2* indicates participants with 2 vascular risk factors; *VRS_3* indicates participants with 3 or more vascular risk factors

Apart from the composite VRS, we also observed significant unique contributions of each individual risk factor (except obesity) to the WMBAG when controlling for other risk factors and covariates, in model 2a (Table 4). Having diabetes would accelerate WM ageing by 1.39 years (WMBAG = 1.39, *p* < 0.001), followed by hypertension (0.87 years, *p* < 0.001) and smoking (0.69 years, *p* < 0.001). Interestingly, in models 2b–f, we found that all interaction terms were not significant, except for the interaction between obesity and sex (Table 4). We found that female participants with obesity did not have larger WMBAG, while males with obesity had significant larger WMBAG than those without obesity (Fig. 4C). Different APOE ε4 carrier status was not associated with the WMBAG in any of the models (all *p* values > 0.05).

**Table 4:** Associations between different vascular risk factors and WMBAG

	Unstandardised beta	95%CI		*p*-value
		Lower bound	Upper bound	
*Main effects (model 2a)*				
Chronological age	− 0.028	− 0.038	− 0.017	< 0.001
Sex	0.052	− 0.099	0.202	0.503
Scanner	0.235	0.149	0.321	< 0.001
APOE status	0.057	− 0.088	0.202	0.440
Hypertension	0.871	0.713	1.028	< 0.001
Diabetes	1.390	1.051	1.729	< 0.001
Hypercholesterolemia	0.311	0.119	0.503	0.002
Obesity	0.161	− 0.033	0.355	0.103
Smoking	0.689	0.537	0.841	< 0.001
*Interactions (models 2b–f)*				
Hypertension * sex	0.112	− 0.183	0.406	0.458
Diabetes * sex	0.318	− 0.278	1.040	0.257
Hypercholesterolemia * sex	− 0.221	− 0.574	0.131	0.219
Obesity * sex	1.023	0.643	1.403	< 0.001
Smoking * sex	0.275	− 0.026	0.576	0.073

Independent main effects of vascular risk factors on WMBAG were analysed by adding all vascular risk factors into the regression model. Interaction effects were analysed by adding each vascular risk factor and its corresponding interaction term to the model

*WMBAG* white matter brain age gap; *APOE* Apolipoprotein E; *CI* confidence interval

#### Association between cognition and WMBAG

After controlling for age, sex, scanner, APOE and education, WMBAG was found to be significantly and negatively associated with baseline processing speed (unstandardised b = − 0.025, *p* < 0.001), executive function (unstandardised b = − 0.018, *p* < 0.001), memory (unstandardised b = − 0.008, *p* = 0.027) and global cognition (unstandardised b = − 0.022, *p* < 0.001). However, only speed, executive function and global cognition survived Bonferroni correction.

Mediation analysis on baseline data is summarised in Supplementary Table e-3. Significant mediation effects of WMBAG were observed for the associations between hypertension and processing speed, executive function and global cognition (bs = − 0.019 to − 0.014, all *p* values < 0.001), and the associations between diabetes and these cognitive outcomes (bs = − 0.033 to − 0.024, all *p* values < 0.001). Smoking was also found to be associated with processing speed and executive function decline via WMBAG (b = − 0.014, *p* < 0.001 and b = − 0.010, *p* < 0.001, respectively), and was also associated with executive function, memory, and global cognition directly (bs = − 0.071 to − 0.007, all *p* values < 0.05. Obesity was associated with processing speed decline directly (b = − 0.103, *p* = 0.001), but not mediated by WMBAG (b = 0.003, *p* = 0.200).

### Longitudinal analysis

The demographics of 1409 participants with baseline and follow-up scans are shown in Table 1. Estimated white matter brain age and WMBAG for both timepoints are presented in Table 2. Generally, participants underwent an average 2.25 ± 0.12 years of follow up (ranging from 2.01 to 2.67 years). One thousand three hundred and fourteen (93.26%) participants had increased white matter brain age with an average of 2.57 ± 1.48 years (dependent t-test, *p* < 0.001) between baseline and follow-up scans (Figure e-2). VRS was not associated with the WMBAG change, no individual vascular risk factors contributed significantly to the WMBAG change except for obesity (Supplementary Table e-4). No significant associations between WMBAG change and cognition change were observed (Supplementary Table e-5). We did not find any significant mediation effect of WMBAG change on the relationships between vascular risk factors and cognition change (all *p* values > 0.05, see Supplementary Table e-6).

## Discussion

This study had three main findings. First, we successfully developed a 3D-CNN deep learning model for estimating brain age based on cerebral white matter only. Second, we found that, cross-sectionally, cerebrovascular risk factors, both individually and collectively, were significantly associated with WMBAG increase; higher WMBAG was associated with poorer cognitive performance, especially processing speed and executive function. Third, participants with diabetes showed the largest WMBAG increase (1.39 years) independently when controlling other risk factors, followed by hypertension (0.87 years) and smoking (0.69 years). Moreover, we demonstrated that WMBAG played a mediation role between vascular risk factors, namely, hypertension and diabetes, and declined cognition, especially with a slower processing speed and declined executive function.

Our trained 3D-CNN model showed a better MAE in age prediction compared with many previous brain age studies [21]. Technically, this model was trained in a large population sample of healthy community-dwelling participants from the UK Biobank, which enabled strong power for model estimation. The combined information extracted from all five DWI-derived maps also improved the model accuracy (Supplementary Table e-2); the information from the fusion of five DWI maps resulted in a lower MAE and higher Pearson’s coefficient. FA, MD, AxD, RD and MO maps are widely recognized DWI feature maps and have been shown to be significantly correlated with vascular risk factors [22, 23]. Each of them taps into distinct physiological properties of the white matter microstructure, from which the deep learning model extracted essential information for white matter brain age prediction. Taking all these technical steps into consideration, our deep learning model for white matter age predication was well established. Moreover, we also validated the model’s performance in the subsample with two time-point scans. After approximately an average of 2.25 years of follow up, 93.26% of the 1409 participants had an increased white matter brain age compared with baseline, which further demonstrated that our deep learning model was robust.

Interestingly, the WMBAG computed in this study correlated with the cerebrovascular burden but not with the neurodegenerative risk factor, namely APOE genotype. In our study, except for obesity, all vascular risk factors were significantly correlated with WMBAG with diabetes and hypertension having the highest correlations. Our results suggested that the participants with diabetes on average had a WMBAG of 1.39-years older than that of the non-diabetic participants; similarly, the brain of a hypertensive participant would have a WMBAG of 0.871 years older than those without hypertension. These findings highlighted the importance of diabetes and hypertension on the white matter health as they are widely reported to be highly associated with morbidity and mortality of cerebrovascular diseases (CVD) [24, 25]. However, no significant association was found between the APOE ε4 allele(s) status and WMBAG in this study. APOE ε4 allele(s) has been recognized as the strongest genetic risk factor for sporadic Alzheimer’s Disease [26]. APOE genotypes with one or two ε4 allele(s) lead to a three to tenfold risk for AD, respectively [27]. While some studies also reported that APOE was correlated with subcortical lesions such as WMH [28] and microbleeds [29], the findings were not always consistent. Most previous brain age studies aimed at capturing the overall changes for the whole brain, therefore are unable to differentiate cerebrovascular burden from neurodegenerative burden. Although increasing evidence has suggested cerebrovascular disease and neurodegenerative disease share multiple risk factors and have overlapping neuropathologies [30, 31], there is a general difference in their MRI manifestations. AD patients usually start grey matter atrophy at premorbid stage and the atrophy progresses with the advance of AD, while those with cerebrovascular disease usually suffer more from the subcortical lesions such as WMH, lacunes, microbleeds and enlarged perivascular spaces [3]. Our study was based on this hypothesis, and we used DWI for white matter brain age computation, given its sensitivity to the microstructural integrity and pathology of subcortical white matter.

Sex dimorphism was observed when we considered the interactive effect between sex and vascular risk factors on the prediction of the WMBAG. Males showed higher WMBAG than females when they had three or more vascular risk factors. Obesity was the only vascular risk factor that showed interactive effect with sex on the WMBAG. Only males with obesity had a significantly greater WMBAG, suggesting that obesity was detrimental to brain ageing in men but not in women. Although the potential mechanisms underlying this sex difference have not been fully understood, other studies including one of our own [32] have also reported this interesting dimorphism, suggesting that females might be more resilient to the detrimental effect of obesity on the brain than males [33, 34]. One hypothesis posits that the distribution of adipose tissues in males and females is different—males tend to accrue more visceral fat, which heightens the vascular burden; conversely women usually accrue more fat in the subcutaneous depot, which is an independent predictor of lower cardiovascular and diabetes-related mortality [35].

Significant associations between WMBAG processing speed, executive function and global cognition after Bonferroni correction were observed cross-sectionally. Processing speed and executive function were considered to be the most vulnerable cognitive domains in CVD [36]. In comparison with AD patients, patients with CVD usually show less pronounced memory deficits [37], although the memory dysfunction may also appear progressively during the later course of the disease. Consistent with the clinical differentiations between AD and CVD, we did not find a significant association between the WMBAG and memory loss, which further demonstrated both specificity and reliability of our white matter brain age model in relation to the cerebrovascular disease burden, and that our model may have clinical utility.

Using mediation analyses in baseline participants, we found that among the five cerebrovascular risk factors, only hypertension and diabetes were associated with processing speed, executive function, and global cognition through the mediation of WMBAG. These findings validated the underlying pathway that the vascular risk factors would contribute to the pathological changes in the white matter and then lead to the cognitive dysfunction, However, our longitudinal analysis did not yield a significant mediation effect of WMBAG change between any vascular risk factor and cognitive decline. This may be partly due to the short period of time between baseline and follow-up (i.e., about 2 years), where significant changes in WMBAG might be too subtle to be detected. Moreover, many participants had better cognition at follow-up than baseline due perhaps to practice effects.

We believe that future work should be carried out to further investigate the relationship between vascular risk factors and white matter brain age. Our stratification for the level of risk factors was based on the number of vascular risk factors, regardless of the type of vascular risk factors a participant had or the specific contribution of each risk factor. For example, a participant with diabetes only would be grouped with anyone with just one of the vascular risk factors we investigated regardless of the type, i.e., any of one of hypertension, diabetes, hypercholesterolemia, obesity or smoking. In this study, we ‘binarised’ our participants into ‘presence’ or ‘absence’ of a vascular risk factor. Comparisons were therefore limited to ‘yes’ or ‘no’ as to whether the participant had that particular vascular risk factor or not, with a lack of more nuanced investigations of the disease stage or disease severity dependent effects of clinical measurements on these risk factors. Additionally, although we have a longitudinal subset with a large sample size from UK Biobank, the follow-up time might be too short to uncover significant brain structural and cognitive changes. Some cerebrovascular and neurodegenerative pathologies may coexist in the brain ageing process, and it is difficult to differentiate the effect of these pathologies on white matter and grey matter distinctively.

## Supplementary Information

Below is the link to the electronic supplementary material.

Supplementary file1 (DOCX 4696 KB)
