# Supplementary material for: White matter brain age as a biomarker of cerebrovascular burden in the ageing brain
Source: Eur Arch Psychiatry Clin Neurosci. 2024 Feb 29;275(8):2203–13. doi: 10.1007/s00406-024-01758-3 (PMC12638326; doi:10.1007/s00406-024-01758-3)
Supplement: Supplementary file 1 — Supplementary file1 (DOCX 4696 KB) [file 406_2024_1758_MOESM1_ESM.docx]

Supplementary Checklist

[Supplementary Methods 1](#_Toc128934310)

[Evaluation of risk factors 2](#_Toc128934311)

[Cognitive tests 3](#_Toc128934312)

[Statistical models 3](#_Toc128934313)

[Supplementary Tables 4](#_Toc128934314)

[Table e-1 UK Biobank exclusive disease codes 4](#_Toc128934315)

[Table e-2 Model performance for different DWI maps. 5](#_Toc128934316)

[Table e-3 Associations between baseline vascular risk factors and cognition mediated by WMBAG 6](#_Toc128934317)

[Table e-4 Associations between different vascular risk factors and WMBAG change 7](#_Toc128934318)

[Table e-5 Associations between WMBAG change and cognition change 8](#_Toc128934319)

[Table e-6 Direct and indirect associations between baseline vascular risk factors and longitudinal cognition change 9](#_Toc128934320)

[Supplementary Figures 10](#_Toc128934321)

[Figure e-1 Scatterplots showing the relationship between chronological age and predicted white matter brain age in the healthy test set (A) and unhealthy test set (B). 10](#_Toc128934322)

[Figure e-2 Longitudinal change of white matter brain age for each participant. 11](#_Toc128934323)

# **Supplementary Methods**

## **Evaluation of risk factors**

An Omron HEM-7015IT device was used to automatically evaluate the seated blood pressure twice; the mean blood pressure for each individual was computed by averaging these two measurements. Participants with hypertension were defined with blood pressure over 140/90 mmHg or using lowering blood pressure medication (Field IDs 6177 and 6153). Participants with diabetes were defined according to the doctor’s diagnosis (Field ID 2443) and anti-diabetes medication (Field IDs 6177 and 6153). Hypercholesterolemia was identified according to the medication information (Field IDs 6177 and 6153). Obesity was defined as body-mass-index (BMI) ≥ 30, which was constructed from ratio of height and weight measured during the initial assessment (Field ID 21001). Smoking was defined as current or previous smoking history (Field ID 20116). DNA from a blood sample of first recruited participants (approximately 50,000) were genotyped in the UK biobank using Affymetrix UK BiLEVE Axiom array; the rest were genotyped with Affymetrix UK Biobank Axiom array^1^. Two APOE coding single nucleotide polymorphisms (SNPs) rs7412 and rs429358 downloaded from the genotyped data were used to determine the APOE genotype ^2^. APOE was considered as the major genetic AD risk factor, however, it was also reported to be associated with cerebrovascular lesions. As a result, APOE ε4 carrier status was also included in this study as a covariate and was classified into three categories based on the number of ε4 alleles, i.e., non-carriers; carriers with one ε4 allele; carriers with two ε4 alleles.

## **Cognitive tests**

Raw scores for these tests were standardised by transforming the raw cognitive scores into z-scores using the healthy reference subsample of UK Biobank at baseline. Specific cognitive domain scores were computed by averaging the corresponding cognitive test scores within a domain and then standardising against the healthy subsample. Global cognition was computed by averaging the scores across all three cognitive domains and again standardising against the healthy subsample.

## **Statistical models**

Model 1a: WMBAG ~ 𝛽_1_VRS_1 + 𝛽_2_VRS_2 + 𝛽_3_VRS_3 + 𝛽_4_Chronological_age + 𝛽_5_ Sex + 𝛽_6_Scanner + 𝛽_7_APOE + ε_1_

Model 1b: WMBAG ~ 𝛽_1_VRS_1 + 𝛽_2_VRS_2 + 𝛽_3_VRS_3 + 𝛽_4_VRS_1×Sex + 𝛽_5_ VRS_2×Sex + 𝛽_6_VRS_3×Sex + 𝛽_7_Chronological_age + 𝛽_8_ Sex + 𝛽_9_ Scanner + 𝛽_10_APOE + ε_1_

Model 2a: WMBAG ~ 𝛽_1_Hypertension + 𝛽_2_Diabetes + 𝛽_3_Hypercholesterolemia + 𝛽_4_Obesity + 𝛽_5_Smoking + 𝛽_6_ Chronological_age + 𝛽_7_Sex + 𝛽_8_Scanner + 𝛽_9_APOE + ε_1_

Model 2b: WMBAG ~ 𝛽_1_Hypertension + 𝛽_2_Hypertension×Sex + 𝛽_3_ Chronological_age + 𝛽_4_Sex + 𝛽_5_Scanner + 𝛽_6_APOE + ε_1_

Model 2c: WMBAG ~ 𝛽_1_Diabetes + 𝛽_2_ Diabetes ×Sex + 𝛽_3_ Chronological_age + 𝛽_4_Sex + 𝛽_5_Scanner + 𝛽_6_APOE + ε_1_

Model 2d: WMBAG ~ 𝛽_1_Hypercholesterolemia + 𝛽_2_ Hypercholesterolemia ×Sex + 𝛽_3_ Chronological_age + 𝛽_4_Sex + 𝛽_5_Scanner + 𝛽_6_APOE + ε_1_

Model 2e: WMBAG ~ 𝛽_1_Obesity + 𝛽_2_Obesity×Sex + 𝛽_3_ Chronological_age + 𝛽_4_Sex + 𝛽_5_Scanner + 𝛽_6_APOE + ε_1_

Model 2f: WMBAG ~ 𝛽_1_Smoking + 𝛽_2_Smoking×Sex + 𝛽_3_ Chronological_age + 𝛽_4_Sex + 𝛽_5_Scanner + 𝛽_6_APOE + ε

# **Supplementary Tables**

## **Table e-1 UK Biobank exclusive disease codes**

| Disease | Code |
| --- | --- |
| Stroke or ischaemic stroke | 1081 |
| Transient ischaemic attack | 1082 |
| Subdural haematoma | 1083 |
| Subarachnoid haemorrhage | 1086 |
| Neurological injury/trauma | 1240 |
| psychological/psychiatric problem | 1243 |
| Infections of the nervous system | 1244 |
| Brain/intracranial abscess | 1245 |
| Encephalitis | 1246 |
| Meningitis | 1247 |
| Guillain-Barré syndrome | 1256 |
| Chronic degenerative neurological | 1258 |
| Motor Neuron Disease | 1259 |
| Multiple Sclerosis | 1261 |
| Parkinson’s disease | 1262 |
| Dementia or Alzheimer’s disease | 1263 |
| Epilepsy | 1264 |
| Head injury | 1266 |
| depression | 1286 |
| schizophrenia | 1289 |
| mania/bipolar disorder/manic depression | 1291 |
| Other demyelinating disease | 1397 |
| Cerebral aneurysm | 1425 |
| Cerebral palsy | 1433 |
| Brain haemorrhage | 1491 |
| Spina bifida | 1524 |
| Ischaemic stroke | 1583 |
| Meningioma (benign) | 1659 |

For the reference of cognition standardisation, the participants in UK Biobank with any diseases listed above were removed.

## **Table e-2 Model performance for different DWI maps.**

|  | Healthy (n = 7769) | | | |  | Unhealthy (n = 3399) | | | |
| --- | --- | --- | --- | --- | --- | --- | --- | --- | --- |
|  | Before bias correction | | After bias correction | |  | Before bias correction | | After bias correction | |
|  | MAE (years) | Pearson’s r | MAE (years) | Pearson’s r |  | MAE (years) | Pearson’s r | MAE (years) | Pearson’s r |
| FA | 2.76 | 0.890 | 3.05 | 0.890 |  | 2.95 | 0.882 | 3.24 | 0.882 |
| MD | 2.77 | 0.884 | 3.16 | 0.884 |  | 3.05 | 0.865 | 3.45 | 0.865 |
| AxD | 2.78 | 0.886 | 3.16 | 0.888 |  | 3.07 | 0.868 | 3.43 | 0.868 |
| RD | 2.73 | 0.886 | 3.13 | 0.886 |  | 2.99 | 0.867 | 3.44 | 0.867 |
| MO | 3.01 | 0.875 | 3.53 | 0.875 |  | 3.21 | 0.848 | 3.74 | 0.848 |
| fusion | 2.51 | 0.908 | 2.75 | 0.908 |  | 2.71 | 0.892 | 3.03 | 0.892 |

This table shows the model performance in healthy and unhealthy test data before and after bias correction. Abbreviations: DWI = diffusion weighted imaging; FA = fractional anisotropy; MD = mean diffusivity; AxD = axial diffusivity; RD = radial diffusivity; MO = anisotropy mode; MAE = mean absolute error; fusion = fusion of all five diffusion weighted maps; Pearson’s r = Pearson’s correlation coefficient.

## **Table e-3 Associations between baseline vascular risk factors and cognition mediated by WMBAG**

|  | Processing speed | | | | Executive | | | | Memory | | | | Global cognition | | | |  |
| --- | --- | --- | --- | --- | --- | --- | --- | --- | --- | --- | --- | --- | --- | --- | --- | --- | --- |
|  | Unstandardised beta | 95%CI | | p-value | Unstandardised beta | 95%CI | | p-value | Unstandardised beta | 95%CI | | p-value | Unstandardised beta | 95%CI | | p-value | |
|  |  | Lower bound | Upper bound |  |  | Lower bound | Upper bound |  |  | Lower bound | Upper bound |  |  | Lower bound | Upper bound |  | |
| ***Direct effects*** |  |  |  |  |  |  |  |  |  |  |  |  |  |  |  |  | |
| Hypertension | -0.020 | -0.078 | 0.003 | 0.400 | 0.011 | -0.041 | 0.060 | 0.690 | -0.026 | -0.081 | 0.030 | 0.330 | -0.015 | -0.063 | 0.030 | 0.550 | |
| Diabetes | -0.018 | -0.132 | 0.100 | 0.750 | -0.035 | -0.160 | 0.090 | 0.590 | -0.070 | -0.197 | 0.060 | 0.290 | -0.051 | -0.175 | 0.070 | 0.400 | |
| Hypercholesterolemia | -0.043 | -0.099 | 0.010 | 0.148 | -0.051 | -0.114 | 0.010 | 0.094 | -0.028 | -0.094 | 0.040 | 0.420 | -0.051 | -0.108 | 0.010 | 0.078 | |
| Obesity | **-0.103** | -0.162 | -0.050 | 0.001 | -0.059 | -0.123 | 0.010 | 0.082 | -0.027 | -0.040 | 0.090 | 0.460 | -0.056 | -0.116 | 0.000 | 0.066 | |
| Smoking | -0.028 | -0.073 | 0.020 | 0.218 | **-0.071** | -0.121 | -0.020 | 0.004 | **-0.071** | -0.122 | -0.020 | 0.005 | **-0.007** | -0.012 | -0.020 | 0.004 | |
| ***Indirect effects mediated through baseline WMBAG*** | | | |  |  |  |  |  |  |  |  |  |  |  |  |  | |
| Hypertension | **-0.019** | -0.026 | -0.001 | < 0.001 | **-0.014** | -0.021 | -0.010 | < 0.001 | -0.005 | -0.012 | 0.000 | 0.140 | **-0.016** | -0.023 | -0.010 | <0.001 | |
| Diabetes | **-0.033** | -0.048 | -0.002 | < 0.001 | -**0.024** | -0.038 | -0.010 | < 0.001 | -0.009 | -0.022 | 0.000 | 0.130 | **-0.027** | -0.042 | -0.020 | <0.001 | |
| Hypercholesterolemia | -0.004 | -0.009 | 0.000 | 0.094 | -0.003 | -0.007 | 0.000 | 0.096 | -0.001 | -0.004 | 0.000 | 0.200 | -0.003 | -0.008 | 0.000 | 0.108 | |
| Obesity | 0.003 | -0.090 | 0.000 | 0.200 | -0.002 | -0.007 | 0.000 | 0.210 | -0.0009 | -0.003 | 0.000 | 0.310 | -0.003 | -0.008 | 0.000 | 0.194 | |
| Smoking | **-0.014** | -0.021 | -0.010 | < 0.001 | **-0.010** | -0.017 | -0.010 | < 0.001 | -0.0001 | -0.001 | 0.000 | 0.758 | **-0.0001** | 0.000 | 0.000 | 0.036 | |

This table shows the mediation effect of baseline WMBAG on the associations between baseline vascular risk factors and baseline cognition. Chronological age, sex, scanner, APOE and education were controlled for all models. Raw p values are reported in this table with bold unstandardised beta indicating statistical significance after Bonferroni correction (corrected α level = 0.0125). Abbreviations: WMBAG = white matter brain age gap; CI = confidence interval.

## **Table e-4 Associations between different vascular risk factors and WMBAG change**

|  |  | Unstandardised beta | 95%CI | | p-value |
| --- | --- | --- | --- | --- | --- |
|  |  |  | Lower bound | Upper bound |  |
| **Main effects** | Baseline chronological age | 0.009 | -0.005 | 0.023 | 0.217 |
|  | Sex | -0.204 | -0.401 | -0.006 | 0.043 |
|  | Scanner | -0.285 | -0.391 | -0.179 | < 0.001 |
|  | APOE status | 0.089 | -0.099 | 0.278 | 0.353 |
|  | Hypertension | 0.181 | -0.025 | 0.387 | 0.085 |
|  | Diabetes | 0.322 | -0.154 | 0.798 | 0.185 |
|  | Hypercholesterolemia | -0.200 | -0.465 | 0.066 | 0.140 |
|  | Obesity | -0.371 | -0.644 | -0.099 | 0.008 |
|  | Smoking | -0.054 | -0.262 | 0.154 | 0.612 |
| **Interactions** | Hypertension*Sex | 0.291 | -0.098 | 0.680 | 0.143 |
|  | Diabetes*Sex | 0.316 | -0.639 | 1.270 | 0.516 |
|  | Hypercholesterolemia*Sex | 0.085 | -0.408 | 0.578 | 0.736 |
|  | Obesity*Sex | -0.012 | -0.542 | 0.517 | 0.964 |
|  | Smoking*Sex | 0.194 | -0.215 | 0.603 | 0.353 |

Independent main effects of vascular risk factors on WMBAG change were analysed by adding all vascular risk factors into the regression model. Interaction effects were analysed by adding each vascular risk factor and its corresponding interaction term to the model. Abbreviations: WMBAG = white matter brain age gap; APOE = Apolipoprotein E; CI = confidence interval.

## **Table e-5 Associations between WMBAG change and cognition change**

|  | Processing speed change | | | | Executive change | | | | Memory change | | | | Global cognition change | | | |
| --- | --- | --- | --- | --- | --- | --- | --- | --- | --- | --- | --- | --- | --- | --- | --- | --- |
|  | Unstandardised beta | 95%CI | | p-value | Unstandardised beta | 95%CI | | p-value | Unstandardised beta | 95%CI | | p-value | Unstandardised beta | 95%CI | | p-value |
|  |  | Lower bound | Upper bound |  |  | Lower bound | Upper bound |  |  | Lower bound | Upper bound |  |  | Lower bound | Upper bound |  |
| Baseline chronological age | -0.006 | -0.012 | < 0.001 | 0.057 | -0.003 | -0.008 | 0.003 | 0.346 | -0.005 | -0.014 | 0.003 | 0.231 | -0.006 | -0.011 | -0.001 | 0.020 |
| Sex | 0.068 | -0.016 | 0.152 | 0.114 | 0.010 | -0.07 | 0.090 | 0.810 | -0.005 | -0.127 | 0.116 | 0.933 | 0.030 | -0.043 | 0.104 | 0.417 |
| Scanner | -0.043 | -0.091 | 0.004 | 0.075 | -0.021 | -0.067 | 0.024 | 0.350 | 0.060 | -0.009 | 0.128 | 0.087 | -0.001 | -0.042 | 0.041 | 0.972 |
| College | 0.024 | -0.059 | 0.108 | 0.567 | 0.052 | -0.028 | 0.132 | 0.202 | -0.053 | -0.174 | 0.068 | 0.394 | 0.014 | -0.059 | 0.087 | 0.703 |
| WMBAG | 0.020 | -0.006 | 0.045 | 0.134 | 0.008 | -0.016 | 0.032 | 0.521 | -0.012 | -0.049 | 0.025 | 0.522 | 0.007 | -0.015 | 0.029 | 0.533 |

This table shows the relationship between WMBAG change and cognition change after controlling for baseline chronological age, sex, scanner, and college. Abbreviations: WMBAG = white matter brain age gap; CI = confidence interval.

## **Table e-6 Direct and indirect associations between baseline vascular risk factors and longitudinal cognition change**

|  | Processing speed change | | |  | Executive change | | |  | Memory change | | |  | Global cognition change | | |  |
| --- | --- | --- | --- | --- | --- | --- | --- | --- | --- | --- | --- | --- | --- | --- | --- | --- |
|  | Unstandardised beta | 95%CI | | p-value | Unstandardised beta | 95%CI | | p-value | Unstandardised beta | 95%CI | | p-value | Unstandardised beta | 95%CI | | p-value |
|  |  | Lower bound | Upper bound |  |  | Lower bound | Upper bound |  |  | Lower bound | Upper bound |  |  | Lower bound | Upper bound |  |
| ***Direct effects*** |  |  |  |  |  |  |  |  |  |  |  |  |  |  |  |  |
| Hypertension | -0.015 | -0.120 | 0.090 | 0.750 | 0.028 | -0.072 | 0.0130 | 0.590 | -0.048 | -0.198 | 0.090 | 0.500 | -0.015 | -0.108 | 0.080 | 0.760 |
| Diabetes | -0.110 | -0.376 | 0.150 | 0.420 | 0.043 | -0.212 | 0.310 | 0.730 | -0.029 | -0.361 | 0.300 | 0.880 | -0.041 | -0.298 | 0.200 | 0.790 |
| Hypercholesterolemia | 0.018 | -0.128 | 0.160 | 0.800 | 0.001 | -0.140 | 0.140 | 0.990 | -0.028 | -0.094 | 0.040 | 0.420 | -0.049 | -0.173 | 0.080 | 0.470 |
| Obesity | -0.091 | -0.045 | 0.220 | 0.180 | -0.001 | -0.127 | 0.130 | 0.970 | -0.039 | -0.241 | 0.170 | 0.700 | 0.021 | -0.093 | 0.130 | 0.730 |
| Smoking | 0.056 | -0.005 | 0.010 | 0.300 | 0.037 | -0.064 | 0.140 | 0.470 | 0.060 | -0.092 | 0.220 | 0.430 | 0.063 | -0.031 | 0.160 | 0.180 |
| ***Indirect effects mediated through WMBAG change*** | | | |  |  |  |  |  |  |  |  |  |  |  |  |  |
| Hypertension | 0.006 | -0.002 | 0.020 | 0.180 | 0.004 | -0.002 | 0.010 | 0.240 | -0.002 | -0.015 | 0.010 | 0.770 | 0.003 | -0.003 | 0.010 | 0.320 |
| Diabetes | 0.014 | -0.005 | 0.040 | 0.190 | 0.010 | -0.007 | 0.030 | 0.260 | -0.004 | -0.037 | 0.030 | 0.780 | 0.009 | -0.008 | 0.030 | 0.330 |
| Hypercholesterolemia | -0.005 | -0.017 | 0.000 | 0.270 | -0.004 | -0.014 | 0.000 | 0.350 | 0.001 | -0.010 | 0.020 | 0.800 | -0.003 | -0.012 | 0.000 | 0.400 |
| Obesity | -0.085 | -0.024 | 0.000 | 0.140 | -0.006 | -0.020 | 0.000 | 0.220 | 0.002 | -0.016 | 0.020 | 0.800 | -0.005 | -0.019 | 0.000 | 0.300 |
| Smoking | 0.001 | -0.005 | 0.010 | 0.760 | 0.001 | -0.005 | 0.010 | 0.790 | -0.0003 | -0.008 | 0.010 | 0.890 | 0.0007 | -0.004 | 0.010 | 0.800 |

This table shows the direct and indirect associations between baseline vascular risk factors and longitudinal cognitive change mediated through WMBAG change. Chronological age, sex, scanner, and education were controlled for all models. Raw p values were reported in this table with bold unstandardised beta unstandardised beta indicating statistical significance after Bonferroni correction. Abbreviations: WMBAG = white matter brain age gap; CI = confidence interval.

# **Supplementary Figures**


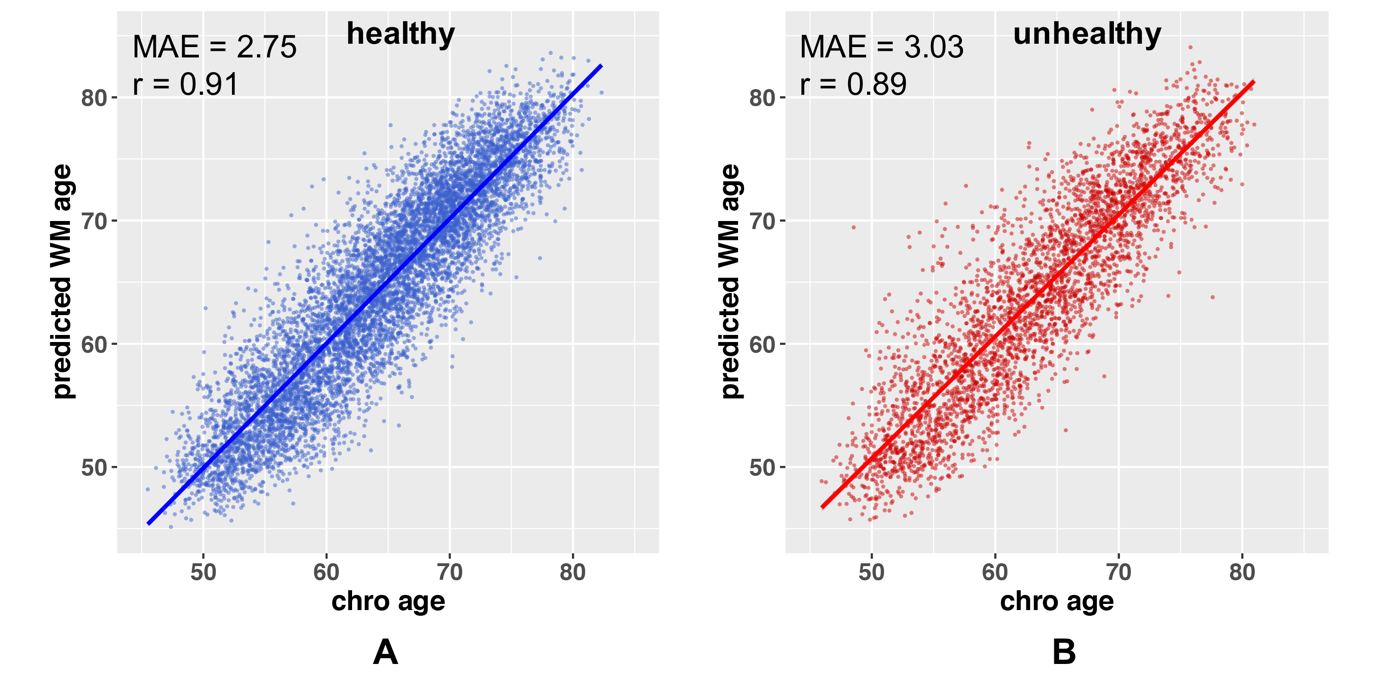


**Figure e-1 Scatterplots showing the relationship between chronological age and predicted white matter brain age in the healthy test set (A) and unhealthy test set (B).** MAE and the correlation coefficient (r) were listed in the upper-left corner of each sub-plot. Abbreviations: r = Pearson’s correlation coefficient; MAE = mean absolute error; WM = white matter; chro age = chronological age.

**
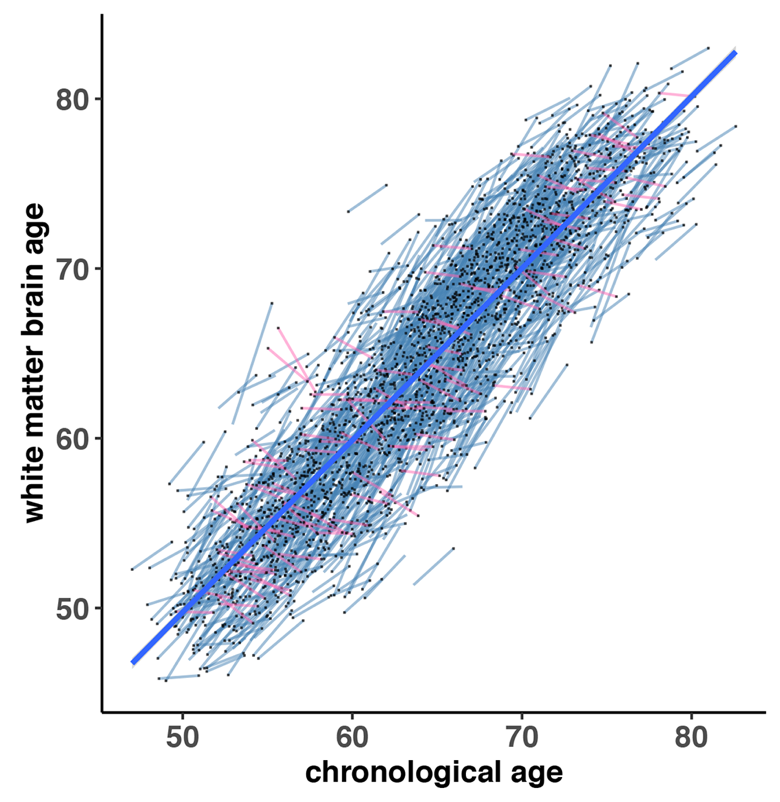
**

**Figure e-2 Longitudinal change of white matter brain age for each participant.** Each participant has two time-point white matter brain ages (shown as two dots), connected with either a short blue line indicating white matter brain age increase (93.26%), or a red line indicating white matter brain age decrease. The bold blue line is a mean line fitted using white matter brain ages from all participants at both timepoints.

1. Bycroft C, Freeman C, Petkova D, Band G, Elliott LT, Sharp K, et al. The uk biobank resource with deep phenotyping and genomic data. Nature. 2018;562:203-209

2. Oldmeadow C, Holliday EG, McEvoy M, Scott R, Kwok JB, Mather K, et al. Concordance between direct and imputed apoe genotypes using 1000 genomes data. J Alzheimers Dis. 2014;42:391-393
